# Supplementary material for: Electric-field-enhanced second-harmonic domain contrast and nonreciprocity in a van der Waals antiferromagnet
Source: Nat Commun. 2024 Aug 30;15:7542. doi: 10.1038/s41467-024-51943-0 (PMC11364783; doi:10.1038/s41467-024-51943-0)
Supplement: Supplementary file 1 — Supplementary Information [file 41467_2024_51943_MOESM1_ESM.pdf]

Supplementary Information for

Electric-field-enhanced second-harmonic domain contrast and nonreciprocity in a van der Waals antiferromagnet

Ziqian Wang[1,*], Meng Wang[1,2], Jannis Lehmann[1,3], Yuki Shiomi[4], Taka-hisa Arima[1,5], Naoto Nagaosa[1], Yoshinori Tokura[1,6,7] and Naoki Ogawa[1]

[1]RIKEN Center for Emergent Matter Science (CEMS), Wako 351-0198, Japan.
[2]School of Integrated Circuits and Electronics, MIIT Key Laboratory for Low-Dimensional Quantum Structure and Devices, Beijing Institute of Technology, Beijing 100081, China.
[3]Department of Physics, ETH Zurich, 8093 Zurich, Switzerland.
[4]Department of Basic Science, University of Tokyo, Tokyo 153-8902, Japan.
[5]Department of Advanced Materials Science, University of Tokyo, Kashiwa 277-8561, Japan.
[6]Department of Applied Physics, University of Tokyo, Tokyo 113-8656, Japan.
[7]Tokyo College, University of Tokyo, Tokyo 113-8656, Japan

*Address correspondence to: ziqian.wang@riken.jp

The Supplementary Information includes:
Supplementary Figs. 1-7
Supplementary Table 1
Supplementary Notes 1-5

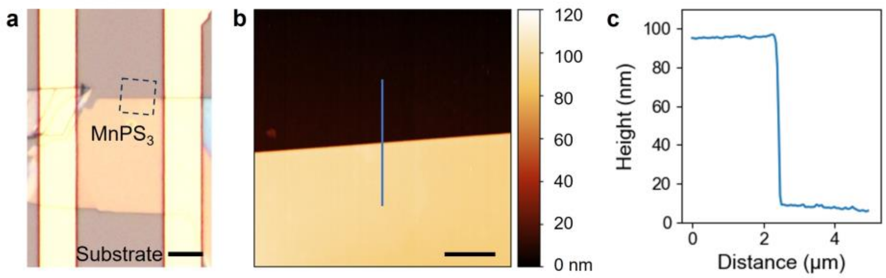

Supplementary Fig. 1 | Sample thickness. a Optical microscopy image with a marked dashed square region near the edge of the flake. Scale bar: 10 μm. **b** Atomic-force-microscopy topography image of the same region. Scale bar: 2 μm. **c** Height profile along the blue line in (**b**), indicating a flake thickness of approximately 90 nm.

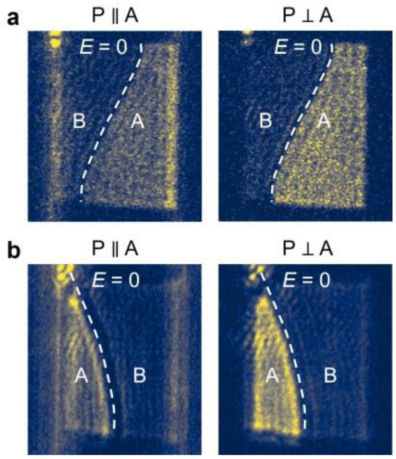

Supplementary Fig. 2 | Variation in domain morphology after different cooling cycles. a,b SHG images acquired after separate cooling events through T_N, at a sample temperature of 10 K using a fundamental wavelength of 840 nm. Domain walls are indicated by white dashed lines for clarity. Images in (**a**) correspond to those in Fig. 2. This observation indicates that the contrast arises from antiferromagnetic 180° domains rather than structural defects like crystallographic twins, which are much less mobile.

Supplementary Note 1. Details on the symmetry-adapted SH intensity formula

We demonstrate that both under the *p*-*d* charge-transfer resonance condition and the *d*-*d* transition resonance condition, the symmetry-adapted SH intensity adheres to the same form as in Eq. (1) in the main text, with variations only in the ingredients of $\chi_{a,b,c}$.

The general source term $\mathbf{S}$ for SHG is given by Eq. (S1)[1,2], and the SH intensity I_{SH} on the detector is proportional to $|\mathbf{S}\cdot\hat{\mathbf{e}}_{\mathrm{A}}|^2$, where $\hat{\mathbf{e}}_{\mathrm{A}}$ represents the analyzer direction.

$$\mathbf{S} = \mu_0\left(\frac{\partial^2\mathbf{P}^{\mathrm{NL}}}{\partial t^2} + \nabla\times\frac{\partial\mathbf{M}^{\mathrm{NL}}}{\partial t}\right) \quad \text{(S1)}$$

Here, $\mathbf{P}^{\mathrm{NL}}$ and $\mathbf{M}^{\mathrm{NL}}$ are the nonlinear electric polarization and magnetization at the frequency 2ω induced by the fundamental light at frequency ω. Their detailed expressions depend on specific resonance conditions as explained below.

A. *p-d* charge-transfer resonance

Under the resonant nonlinear interaction described in Fig. 1b in the main text, e.g., using a fundamental wavelength of 840 nm, the primary ED and MD SHG processes are given by Eqs. (S2) and (S3), respectively.

$$P_i^{\mathrm{eee}}(2\omega) = \varepsilon_0 \chi_{ijk}^{\mathrm{eee}} E_j(\omega) E_k(\omega) \quad \text{(S2)}$$

$$P_i^{\mathrm{eem}}(2\omega) = \frac{\varepsilon_0}{c} \chi_{ijk}^{\mathrm{eem}} E_j(\omega) H_k(\omega) \quad \text{(S3)}$$

Here, ε_0 is the permittivity of free space and c is the speed of light. They both factor into $\mathbf{P}^{\mathrm{NL}}$ in Eq. (S1), with no contribution to $\mathbf{M}^{\mathrm{NL}}$. In Eq. (S3), the electric and magnetic field components of the fundamental light, propagating along z, are linked by $H_k(\omega) = (1/c\mu_0)\epsilon_{kzk'} E_{k'}(\omega)$, where ϵ_{ijk} is the Levi-Civita symbol, and $i, j, k,$ and k' can each take on x or y. For transmission SHG with both fundamental and SH wavevectors being parallel to z, the active $\chi_{ijk}^{\mathrm{eee}}$ and $\chi_{ijk}^{\mathrm{eem}}$ tensor components are listed below, common to magnetic space groups $\mathrm{C}\,2'/\mathrm{m}$ and Cm[3].

$$\chi_{ijk}^{\mathrm{eee}}: \quad \chi_{xxx}^{\mathrm{eee}}, \chi_{xyy}^{\mathrm{eee}}, \chi_{yxy}^{\mathrm{eee}} = \chi_{yyx}^{\mathrm{eee}} \quad \text{(S4)}$$

$$\chi_{ijk}^{\mathrm{eem}}: \quad \chi_{xxy}^{\mathrm{eem}}, \chi_{xyx}^{\mathrm{eem}}, \chi_{yxx}^{\mathrm{eem}}, \chi_{yyy}^{\mathrm{eem}} \quad \text{(S5)}$$

Combining Eqs. (S1) to (S5), the polarization-dependent SH intensity becomes

$$I_{\mathrm{SH}} \propto \begin{cases} |(\chi_a \cos^2\varphi + \chi_b \sin^2\varphi)\cos\varphi + \chi_c \sin 2\varphi \sin\varphi|^2 & \mathrm{P} \parallel \mathrm{A} \\ |(\chi_a \cos^2\varphi + \chi_b \sin^2\varphi)\sin\varphi - \chi_c \sin 2\varphi \cos\varphi|^2 & \mathrm{P} \perp \mathrm{A}' \end{cases}$$

reproducing Eq. (1) in the main text, with $\chi_a = \chi_{xxy}^{\mathrm{eem}} + \chi_{xxx}^{\mathrm{eee}}$, $\chi_b = -\chi_{xyx}^{\mathrm{eem}} + \chi_{xyy}^{\mathrm{eee}}$, and $\chi_c = (\chi_{yyy}^{\mathrm{eem}} - \chi_{yxx}^{\mathrm{eem}})/2 + \chi_{yxy}^{\mathrm{eee}}$. Note that the electric-field dependences of $\chi_{ijk}^{\mathrm{eee}}$ is implicitly included here, accounting for the ΔED term. Explicitly writing the ΔED terms down yields $\chi_a = \chi_{xxy}^{\mathrm{eem}} + \chi_{xxx}^{\mathrm{eee}} + \Delta\chi_{xxx}^{\mathrm{eee}}$, $\chi_b = -\chi_{xyx}^{\mathrm{eem}} + \chi_{xyy}^{\mathrm{eee}} + \Delta\chi_{xyy}^{\mathrm{eee}}$, and $\chi_c = (\chi_{yyy}^{\mathrm{eem}} - \chi_{yxx}^{\mathrm{eem}})/2 + \chi_{yxy}^{\mathrm{eee}} + \Delta\chi_{yxy}^{\mathrm{eee}}$, as presented in the main text, where e/m represents eee/eem in the superscript.

B. *d-d* transition resonance

When the SH photon energy resonates with a typically ED-forbidden *d-d* transition, e.g., the case with fundamental wavelength of 920 nm (Supplementary Fig. 5a), the SH photon emission process primarily exhibits a strong MD character, resulting in dominant MD SHG described by Eq. (S6), with a concurrent weaker ED SHG component following the same form as in Eq. (S2).

$$M_i^{\mathrm{mee}}(2\omega) = \frac{\varepsilon_0 c}{n} \chi_{ijk}^{\mathrm{mee}} E_j(\omega) E_k(\omega) \quad \text{(S6)}$$

Here, n represents the refractive index of the material for the SH light. The $M_i^{\mathrm{mee}}(2\omega)$ and $P_i^{\mathrm{eee}}(2\omega)$ give rise to $\mathbf{M}^{\mathrm{NL}}$ and $\mathbf{P}^{\mathrm{NL}}$ in Eq. (S1), respectively. The active $\chi_{ijk}^{\mathrm{mee}}$ tensor components, for light incident along z, are listed below, and active $\chi_{ijk}^{\mathrm{eee}}$ are identical to Eq. (S4). These tensor forms are again common to magnetic space groups $\mathrm{C}\,2'/\mathrm{m}$ and Cm[3].

$$\chi_{ijk}^{\mathrm{mee}}: \quad \chi_{yxx}^{\mathrm{mee}}, \chi_{yyy}^{\mathrm{mee}}, \chi_{xxy}^{\mathrm{mee}} = \chi_{xyx}^{\mathrm{mee}} \quad \text{(S7)}$$

By inserting Eqs. (S2), (S4), (S6), and (S7) into Eq. (S1), the polarization-dependent SH intensity takes the same form as in Eq. (1) in the main text, but with $\chi_a = \chi_{yxx}^{\text{mee}} + \chi_{xxx}^{\text{eee}}$, $\chi_b = \chi_{yyy}^{\text{mee}} + \chi_{xyy}^{\text{eee}}$, and $\chi_c = -\chi_{xxy}^{\text{mee}} + \chi_{yxy}^{\text{eee}}$. Again, χ_{ijk}^{eee} implicitly includes the field-dependent ΔED contributions, resulting in $\chi_a = \chi_{yxx}^{\text{mee}} + \chi_{xxx}^{\text{eee}} + \Delta\chi_{xxx}^{\text{eee}}$, $\chi_b = \chi_{yyy}^{\text{mee}} + \chi_{xyy}^{\text{eee}} + \Delta\chi_{xyy}^{\text{eee}}$, and $\chi_c = -\chi_{xxy}^{\text{mee}} + \chi_{yxy}^{\text{eee}} + \Delta\chi_{yxy}^{\text{eee}}$.

Overall, Eq. (1), as along with the $\chi_{a,b,c}$ analyses (Fig. 4 in the main text, Supplementary Fig. 3 for *p*-*d* charge-transfer resonance, and Supplementary Fig. 6 for *d*-*d* transition resonance), provides a consistent framework for addressing the ED-MD interference across various resonance conditions discussed here.

Supplementary Note 2. Additional notes on the fitting analyses

The complex $\chi_{a,b,c}$ values in Figs. 4a and 4b in the main text were obtained through a two-step fitting analysis. In the first step, for each domain and field condition, the P ∥ A and P ⊥ A SHG-RA patterns were fitted to the simultaneous equation Eq. (1) in the main text. This allows for the determination, for each domain, of complex $\chi_a(E)$, $\chi_b(E)$, and $\chi_c(E)$, with phases referenced to a common value at each E, such as the phases of $\chi_c(E)$ herein, as shown in Supplementary Figs. 3a-d. In the second step, despite the absolute phases of $\chi_c(E)$ being lost for individual E, the relative phase shifts of $\chi_c(E)$ due to E can be retrieved by leveraging the simultaneous linear E dependence of the ΔED components in all $\chi_{a,b,c}$. Specifically, by introducing common phase shifts $\theta_0(E)$ to $\chi_a(E)$, $\chi_b(E)$, and $\chi_c(E)$, which may vary for different E's, all $\chi_{a,b,c}$ are expected to concurrently form straight lines on the complex plane. Figs. 4a and 4b in the main text display the outcome of this fitting, with phases referenced to that of $\chi_c(E = 0)$ for each domain. Note that despite the potential phase difference in $\chi_c(E = 0)$ between the two domains, the ability to achieve nearly antiparallel $\chi_{a,b,c}$ trajectories with similar lengths and simultaneous negated zero-field values (empty circles) by appropriately rotating Figs. 4a and 4b confirms the validity of the proposed scheme in Figs. 3d and 3c in the main text.

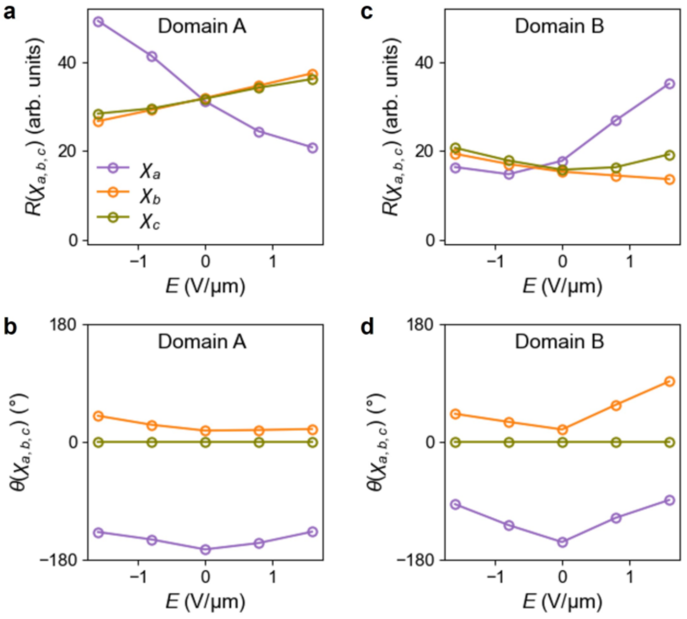

Supplementary Fig. 3 | Amplitudes and phases of $\chi_{a,b,c}$ in the |ED| > |MD| regime (for a fundamental wavelength at 840 nm). a,**b** Electric-field dependence of amplitudes (**a**) and phases (**b**) of $\chi_{a,b,c}$ for Domain A. Phases of $\chi_{a,b}$ are plotted relative to that of χ_c at each field. A second fitting by imposing the constraint that $\chi_{a,b,c}(E)$ have linear E dependences at the same time gives the results as presented in Fig. 4a in the main text. **c**,**d** Replication of (**a**,**b**) for B domain, associated with Fig. 4b in the main text after application of the same fitting procedure. Legends are common to (**a-d**).

Supplementary Note 3. Additional results for the |MD| > |ED| regime at a *d-d* transition resonance.

Supplementary Figs. 4-6 illustrate the |MD| > |ED| regime at a *d-d* transition resonance, as exemplified with a 920 nm fundamental wavelength. For each $\chi_{a,b,c}$, the $E = 0$ response (the empty symbol or approximately the midpoint of the arrow) lies in the same quadrants for Domains A and B in Supplementary Figs. 6c and 6f, in line with the Point 3 in Supplementary Figs. 4b and 4a. This trend contrasts with those lying in opposite quadrants in Figs. 4a and 4b and Figs. 3d and 3c in the main text for the *p-d* charge-transfer resonance. Moreover, for each $\chi_{a,b,c}$, the arrows for Domains A and B in Supplementary Figs. 6c and 6f, representing the trace of ΔED contributions, are nearly parallel and of similar lengths, reproducing the situation depicted in Supplementary Figs. 4a and 4b. Overall, both the *p-d* charge-transfer resonance (|ED| > |MD| regime) in the main text and the *d-d* transition resonance (|MD| > |ED| regime) as described here are well explained within our three-term interference framework.

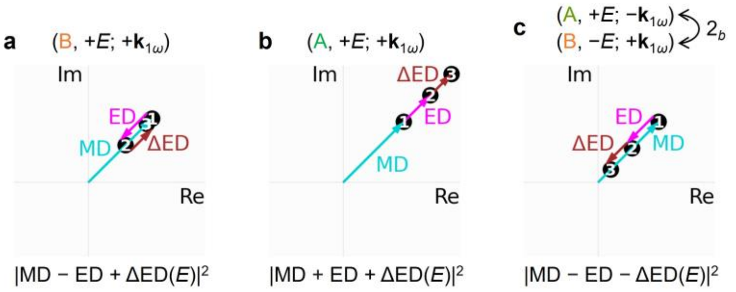

Supplementary Fig. 4 | Conceptual illustration of interfering MD, ED and ΔED transitions in the |MD| > |ED| regime (e.g., for a fundamental wavelength of 920 nm). a-c Conceptual illustration of MD, ED and field-dependent ΔED transitions interfering on the complex plane (counterparts of Figs. 3c-e for the |ED| > |MD| regime in the main text). End points 1 to 3 provide guidance for vector summation in the interference process. The distance between Point 3 and the origin represents the SH amplitude. Point 3 coincides with Point 2 at $E = 0$. The two labels in (**c**) denote the same physical process viewed from front and back. The distinction between (**a**) and (**b**) accounts for domain contrast, while the difference between (**b**) and (**c**) is associated with nonreciprocity.

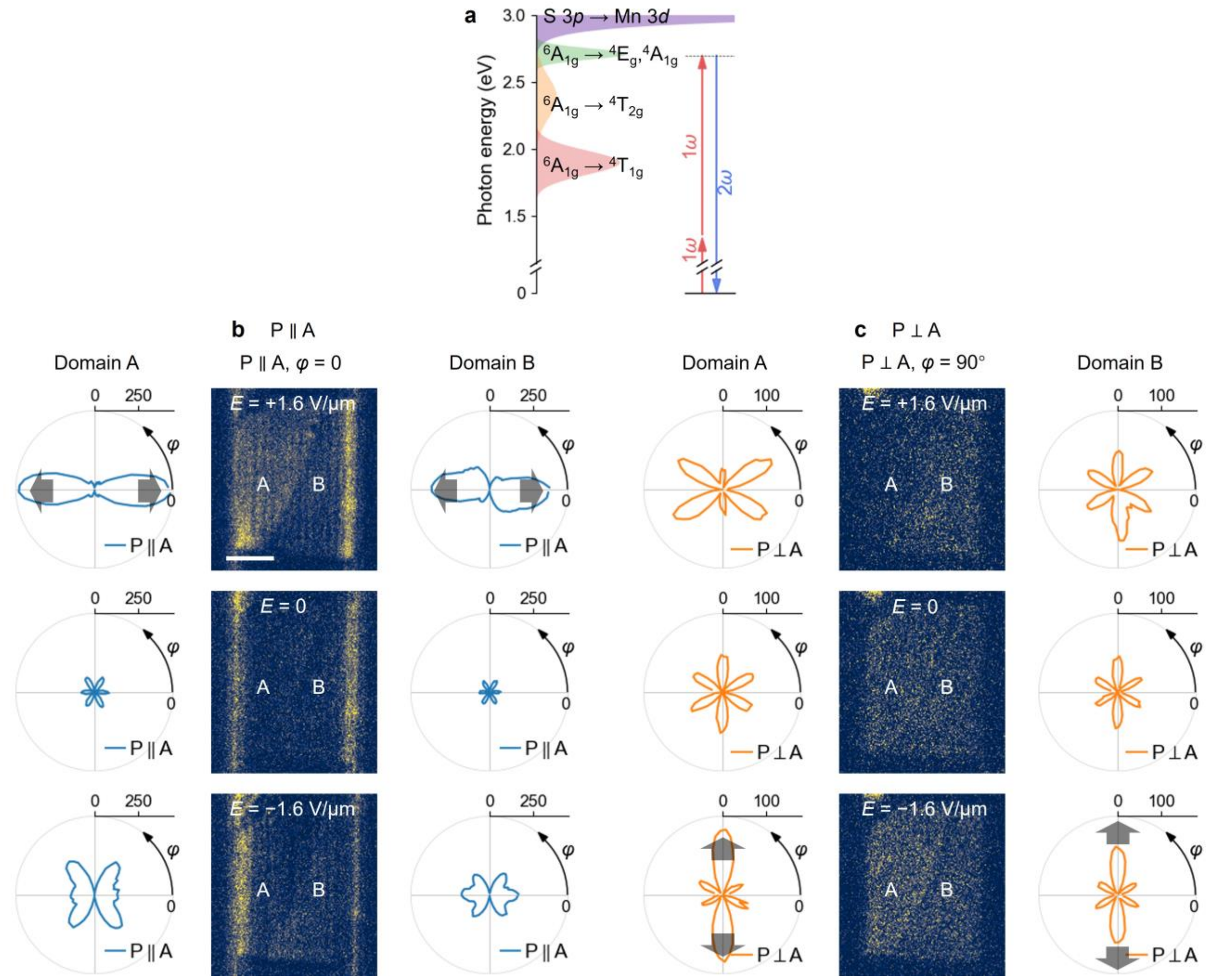

Supplementary Fig. 5 | Electrical control of SHG domain contrast in the |MD| > |ED| regime (for a fundamental wavelength of 920 nm). a Optical energy diagram with photon energy 2ω in resonance with a *d*-*d* transition. **b**,**c** Co- (P ∥ A) and cross- (P ⊥ A) polarization configurations (P: polarizer, and A: analyzer). Middle columns in (**b**,**c**) display SHG images with controlled domain contrast acquired at 10 K under the application of electric fields. Scalebar: 10 μm (shared by all images). Corresponding SHG-RA patterns for Domains A and B appear on the left and right sides of the images, respectively. In contrast to the opposite field dependences observed for different domains in the *p*-*d* charge-transfer resonance case (Fig. 2 in the main text), the electric field dependence here shows similar intensity modulation for Domains A and B, as indicated by the grey arrows, consistent with the interfering scenarios in Supplementary Figs. 4b and 4a.

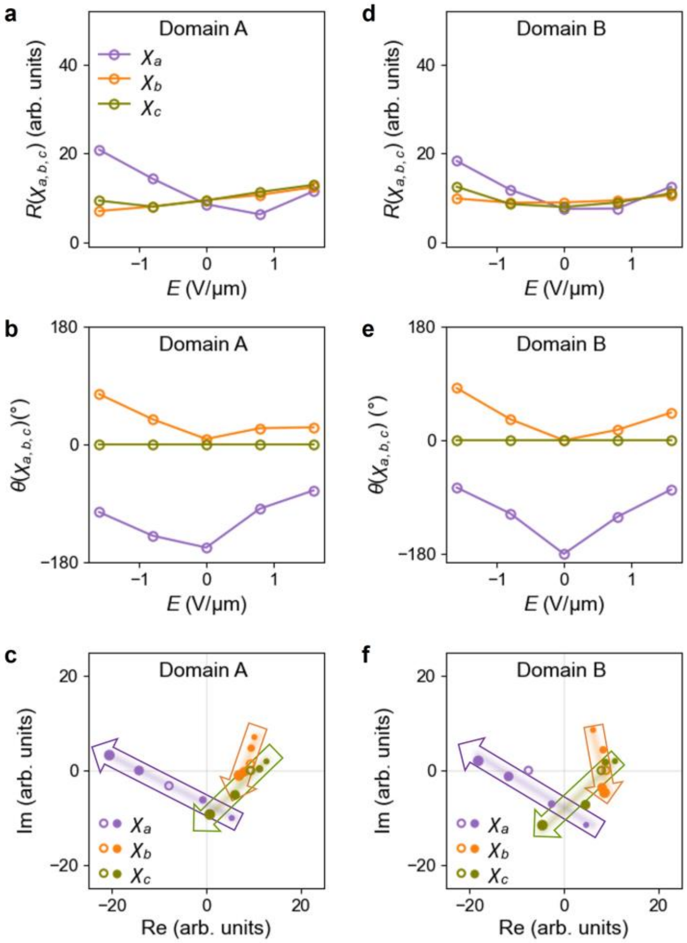

Supplementary Fig. 6 | Variation of complex nonlinear susceptibilities under electric fields in the |MD| > |ED| regime (for a fundamental wavelength at 920 nm). a,**b** Electric field dependence of amplitudes (**a**) and phases (**b**) of $\chi_{a,b,c}$ for Domain A. Phases of $\chi_{a,b}$ are plotted relative to that of χ_c at each field. **c** $\chi_{a,b,c}$ plotted on the complex plane, with shadings and arrows indicating their evolution trends as the electric field increases from negative to positive. Empty symbols represent χ's at zero field. Complex $\chi_{a,b,c}$ values are faithful up to a common phase rotation. Phase of χ_c at $E = 0$ was used as the reference. **d-f** Replicating (**a-c**) for Domain B. (**a-d**) share the same legends.

Supplementary Note 4. Additional notes on the interaction-symmetry-preserved and interaction-symmetry-non-preserved types of electrical control.

Electric field application along the a- and b-axes of $MnPS_3$ exemplifies the interaction-symmetry-preserved and interaction-symmetry-non-preserved scenarios of electrical modulation on SHG, respectively.

An electric field along the a-axis transforms the magnetic point group $2'/\mathrm{m}$ to its unitary subgroup m. In this configuration, the ΔED contribution arises as modifications to the active elements in the zero-field ED SHG tensor χ^{e}_{ijk}, highlighted in red, thereby preserving the SHG interaction symmetry (Supplementary Fig. 7c, left and middle panels). As a result, ΔED SHG emerges along the symmetry-allowed directions of zero-field SHG, as depicted in Supplementary Fig. 7a. The ΔED SHG, represented schematically by the

red curves, interferes with zero-field SHG (right panels), giving rise to the observed finite-field SHG-RA patterns (left panels). Since the maxima of ΔED and zero-field SHG are aligned, the ED-MD-ΔED interference is maximally effective from the symmetry viewpoint. Consequently, we propose such interaction-symmetry-preserved modulation as a general approach for electrical control of SHG domain contrast and nonreciprocity.

In contrast, for an electric field applied along the b-axis, the magnetic point group is transformed to 2′, a polar subgroup distinct from the unitary one. In this scenario, the electric field activates new elements in the tensor χ^{e}_{ijk}, highlighted in red, thus not preserving the SHG interaction symmetry (Supplementary Fig. 7c, middle and right panels). This results in ΔED SHG aligning with symmetry-forbidden directions of zero-field SHG, as illustrated in Supplementary Fig. 7b. Specifically, at 90 and 270° for P ∥ A and 0 and 180° for P ⊥ A, the absence of intensity in the zero-field SHG renders it unsuitable for electrical control of domain contrast and nonreciprocity. Additionally, it is worth noting that lobes at polarization angles of 60, 120, 240, and 300° for P ∥ A, and 30, 150, 210, and 330° for P ⊥ A, are not inherently expected from C2′/m or C2′. Their targeted modulation would be a case-by-case scenario, in addition to the proposed general strategy of interaction-symmetry-preserved type control.

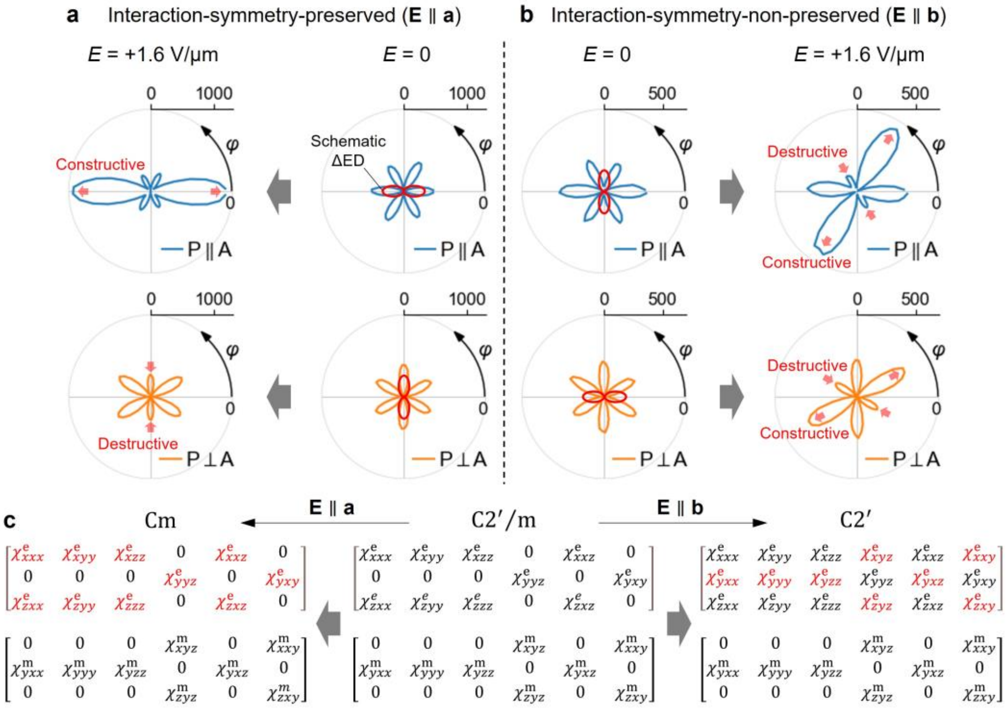

Supplementary Fig. 7 | Comparison of interference scenarios under electric fields applied along two different crystal axes. a SHG-RA patterns with (right) and without (left) application of an electric field along the a-axis, exemplifying an interaction-symmetry-preserved scenario. These patterns correspond to those for Domain A at 10 K in Fig. 2a in the main text. **b** SHG-RA patterns for electric field application along the b-axis, obtained from a different specimen, exemplifying an interaction-symmetry-non-preserved scenario. The red curves in (**a**) and (**b**) schematically represent the pure electric-field-induced contributions (ΔED), which interfere with zero-field SHG to produce finite-field SHG-RA patterns in constructive and destructive manners, as indicated by the red arrows. **c** SHG tensor forms for magnetic groups C2′/m, Cm, and C2′ under $\mathbf{E} = \mathbf{0}$, **E** ∥ **a**, and **E** ∥ **b**, respectively. Upper and lower represent χ^{e}_{ijk} for ED SHG and χ^{m}_{ijk}

for MD SHG, respectively. Elements modulated or activated by an electric field are shown in red in the tensors for Cm or C2′.

Supplementary Note 5. Symmetry for the electrical control of SHG domain contrast and nonreciprocity.

Table S1 lists the candidate magnetic point groups (MPGs) for the proposed effective electrical control of SHG domain contrast and nonreciprocity. These MPGs have $\mathcal{PT}$-symmetry, and the subgroups under electric field coincide with the unitary subgroups of the MPGs. The electric field maintains the ED and MD SHG tensor forms, ensuring the electric-field modulation (ΔED) on existing tensor elements. Due to the invariance of tensor forms under the applied electric field, the SHG yield for, in principle, arbitrary fundamental-light directions can be actively controlled. Antiferromagnet $MnPS_3$ in this study corresponds to the parent group $2'/\mathrm{m}$.

Note that the omission of index φ in the definition of nonreciprocity $\eta(E)$ for $MnPS_3$ (Eq. (3)) is due to the equivalence of SH intensity $I^{\mathrm{A/B},\pm E;\pm k}$ for φ and $-\varphi$ under mirror symmetry. The expression of nonreciprocity may vary with specific MPGs, involving φ and $-\varphi$ as indices in $I^{\mathrm{A/B},\pm E;\pm k,\pm\varphi}$ that comprises $\eta(E)$. Note also that constraining fundamental light incidence along a specific crystal axis expands the list of candidate MPGs, allowing for electrical modulation on a subset of existing tensor elements.

Supplementary Table 1. Candidate MPGs for electrical control of SHG domain contrast and nonreciprocity, along with required electric-field directions and resultant subgroups. The left column lists the MPGs that allow the interference scenarios discussed in Figs. 3c-e in the main text or Supplementary Figs. 4a-c. The middle column specifies the preferred electric field direction for interaction-symmetry-preserved type domain-contrast and nonreciprocity control. The right column indicates the resulting unitary subgroups under the applied field.

MPG (Parent group)	Electric field direction	Subgroup ($E \neq 0$)
$\bar{1}'$	arbitrary	1
$2/\mathrm{m}'$	$E \parallel 2$	2
$2'/\mathrm{m}$	$E \parallel m$	m
$6/\mathrm{m}'$	$E \parallel 6$	6
$4/\mathrm{m}'$	$E \parallel 4$	4
$6/\mathrm{m}'\mathrm{mm}$	$E \parallel 6$	6mm
$4/\mathrm{m}'\mathrm{mm}$	$E \parallel 4$	4mm
$\bar{3}'$	$E \parallel 3$	3
$\bar{3}'\mathrm{m}$	$E \parallel 3$	3m

References

1. Fiebig, M., Fröhlich, D., Krichevtsov, B. B. & Pisarev, R. V. Second Harmonic Generation and Magnetic-Dipole-Electric-Dipole Interference in Antiferromagnetic Cr_2O_3. *Phys. Rev. Lett.* **73**, 2127–2130 (1994).

2. Fiebig, M., Pavlov, V. V. & Pisarev, R. V. Second-harmonic generation as a tool for studying electronic and magnetic structures of crystals: review. *J. Opt. Soc. Am. B* **22**, 96 (2005).

3. Gallego, S. V., Etxebarria, J., Elcoro, L., Tasci, E. S. & Perez-Mato, J. M. Automatic calculation of symmetry-adapted tensors in magnetic and non-magnetic materials: A new tool of the bilbao crystallographic server. *Acta Crystallogr. Sect. A Found. Adv.* **75**, 438–447 (2019).
